# A Novel Rose Hip Preparation with Enhanced Anti-Inflammatory and Chondroprotective Effects

**DOI:** 10.1155/2014/105710

**Published:** 2014-10-13

**Authors:** Joseph Schwager, Nathalie Richard, Rotraut Schoop, Swen Wolfram

**Affiliations:** DSM Nutritional Products Ltd., Department of Human Nutrition & Health, P.O. Box 2676, 4002 Basel, Switzerland

## Abstract

Rose hip powder (RHP) alleviates osteoarthritis (OA) due to its anti-inflammatory and cartilage-protective properties. Substances contained in RHP might contribute to its clinical efficacy. The activity of two RHP (i.e., RH-A, from the whole fruit, RH-B, from fruits without seeds) was investigated in human peripheral blood leukocytes (PBL) and primary chondrocytes (NHAC-kn). RH-A and RH-B diminished the secretion of chemokines and cytokines in LPS/IFN-*γ*-activated PBL, including CCL5/RANTES, CXCL10/IP-10, interleukin- (IL-) 6, and IL-12. Most effects were transcriptional, since gene expression levels were significantly influenced by RH-A and RH-B. In IL-1*β* treated normal chondrocytes (NHAC-kn), both RH preparations reduced the expression of matrix metalloproteinase- (MMP-) 1, MMP-3, and MMP-13 and ADAMTS-4. These changes are associated with diminished inflammatory damage or cartilage erosion. Principal component analysis revealed that (1) RH-A and RH-B modified a large pattern of biomarkers, and (2) RH-B outperformed RH-A. Furthermore, RH-B contained more chondroprotective and anti-inflammatory constituents than RH-A. Thus, RHP contributed to restore cellular homeostasis in PBL and chondrocytes. RH preparations from fruits without seeds are thus expected to have an improved OA-preventive or OA-therapeutic profile, as subsequently shown in a related clinical trial.

## 1. Introduction

Osteoarthritis (OA) reflects the degradation and erosion of the extracellular matrix (ECM) and the subsequent narrowing of space in joints. The changes in ECM structure are due to the activation of enzymatic systems, that is, matrix metalloproteinase (MMPs) and aggrecanase in chondrocytes and synoviocytes [1, 2]. The proinflammatory interleukin- (IL-) 1*β* has a key role in inducing the OA phenotype in chondrocytes [3]. Likewise, nitric oxide (NO) production also correlates with pathophysiological changes in chondrocytes [4–8]. IL-1*β*-activated chondrocytes produce a variety of chemokines [9–12]; this might reflect the implication of cell recruitment during inflammatory processes in OA diseases.

Natural substances reportedly attenuate or delay the onset and progression of OA. Glucosamine and chondroitin have been the most promising substances so far identified, although their effectiveness is a matter of debate. Clinical studies have demonstrated a beneficial effect of rose hip powder (RHP) in the treatment of OA [13–15] (for reviews see [16, 17]). One of its constituents, galacto-lipid (2S)-1, 2-di-O-[(9Z, 12Z, 15Z)-octadeca-9, 12, 15-trienoyl]-3-O-*β*-D-galacto-pyranosyl glycerol (GLGPG), has been found to inhibit chemotaxis of neutrophils [13, 14] and thus could impair pathophysiological cell recruitment to OA lesions. Other RHP constituents such as ascorbic acid, polyphenols, flavonoids, and unsaturated fatty acids might contribute to alleviate OA mainly via their anti-inflammatory properties. Indeed, RHP extracts and unsaturated fatty acids inhibited cyclooxygenase- (COX-) 1 and COX-2 activity [18, 19] and associated PGE_2_ production [20]. In a previous* in vitro* study, the multiple effects of RHP on the production of inflammatory mediators by peripheral blood leukocyte and anabolic and catabolic processes in chondrocytes have been described [20]. The current study aimed at the identification of biological activities of different parts of the rose hip fruit and an improved use of rose hip preparations in the management of OA conditions.

## 2. Materials and Methods

### 2.1. Rose Hip Preparations and Reagents

RHP was prepared from* Rosa canina* and provided by Axellus, Ishøj, Denmark; RH-A consists of dried rose hip powder as described previously [20]; RH-B was prepared from dried rose hip, where the seeds had been removed before the preparation of the powder. The contents in main constituents (see Table 1) have been measured by standard procedures implemented at the Analytical Research Center, DSM Nutritional Products, Kaiseraugst (Switzerland). Briefly, betulinic acid, oleanolic acid, and ursolic acid were determined according to validated in-house methods (available on request); vitamin C and vitamin E were analyzed according to official methods EN14130 and EN12822, respectively; linoleic acid, EPA, and DHA were measured according to the official method of ISO 12966-2. RHP solutions were prepared in DMSO and added to the culture medium concomitantly with the stimulating agent.* E. coli *lipopolysaccharide (LPS, serotype 055:B5) and fetal bovine serum (FBS) were from Sigma/Aldrich (Saint-Louis, MO). RPMI 1640, DMEM, 2-mercaptoethanol, and MEM nonessential amino acids (NEAA) were from Invitrogen (Carlsbad, CA). Human IL-1*β* and recombinant interferon-*γ* (IFN-*γ*) were from PeproTech EC (London, UK).

### 2.2. Cell Culture

Human peripheral blood leukocytes (PBL) and primary chondrocytes from healthy individuals have been cultured and treated with inflammatory stimuli as described [20–22]. Human primary cell culture protocols and* in vitro *peripheral blood leukocyte experiments were approved by the Swiss Federal Office of Public Health (no. A050573/2) and the Ethical Commission of the Kanton Aargau, Switzerland. PBL were obtained from healthy donors. PBL (at 8 × 10^6^ viable cells/mL) were cultured for 12–24 h in phenol-red free RPMI 1640 (containing 0.25% FBS, 0.1 mM NEAA, 50 U/mL penicillin, 50 *μ*g/mL streptomycin, and 50 *μ*M 2-mercaptoethanol) and stimulated with LPS (100 ng/mL) and IFN-*γ* (20 U/mL) with graded amounts of test substances. Normal human articular chondrocytes from knee (NHAC-kn) were seeded into 6-well plates at 0.5 × 10^6^ cells per well and, where indicated, activated with 10 ng/mL IL-1*β* in supplemented CBM (Lonza, Walkersville, MD) in the presence of graded amounts of test compounds for 4 h. In all cell cultures, vehicle (i.e., DMSO) was included at 0.5% final concentration.

For molecular analysis, NHAC-kn cells and PBLs were lysed in RLT buffer (Qiagen, Hilden, Germany) after 4 and 12 h of culture, respectively, and total RNA was extracted. For the analysis of secreted mediators and proteins, PBLs were cultured for 24 h; supernatants were collected and stored at −80°C until use for analysis.

### 2.3. RNA Isolation, cDNA Synthesis, and RT-PCR

The isolation of total RNA, synthesis of cDNA and quantitative RT-PCR has been performed as detailed before [20].

### 2.4. Multiparametric Analysis of Cytokines, Chemokines, and Interleukins

Multiparametric kits were purchased from BIO-RAD Laboratories (Hercules, CA) and used in the LiquiChip Workstation IS 200 (Qiagen, Hilden, Germany) to measure the amount of secreted proteins. Data evaluation was done using the LiquiChip Analyser software (Qiagen).

### 2.5. Statistical Analysis

Data were evaluated by statistical tools described previously [20].* P* values < 0.05 (obtained by using Student's* t* test or one-way ANOVA) were considered to reflect statistically significant differences. Statistical differences between treatment groups were evaluated by the Student's* t*-test. Principal component analysis (PCA) was performed with the package “chemometrics” [23] of the statistical software [24] using the nipals algorithm with centered and scaled input data.

## 3. Results 

### 3.1. Composition of RH-A and RH-B

RH-B, prepared from rose hip fruits after removal of seeds, displayed more potent anti-inflammatory effects. The distinct biological activity of RH-A and RH-B correlated with different contents of constituents: RH-B contained higher amounts of ursolic acid, betulinic acid, GLGPG, and DHA (Table 1). Of those, ursolic acid and betulinic acid and 3-omega PUFAs, but not *β*-carotene, vitamin C, or vitamin E, displayed anti-inflammatory effects (our unpublished data and [25, 26]). Removal of seeds increased the contents of *α*-tocopherol and GLGPG; the latter had anti-inflammatory properties [20]. Other constituents (e.g., vitamin C) were homogenously distributed in rose hip fruit parts.

### 3.2. Effect of RH-A and RH-B on Peripheral Blood Leukocytes

**Figure 1:**
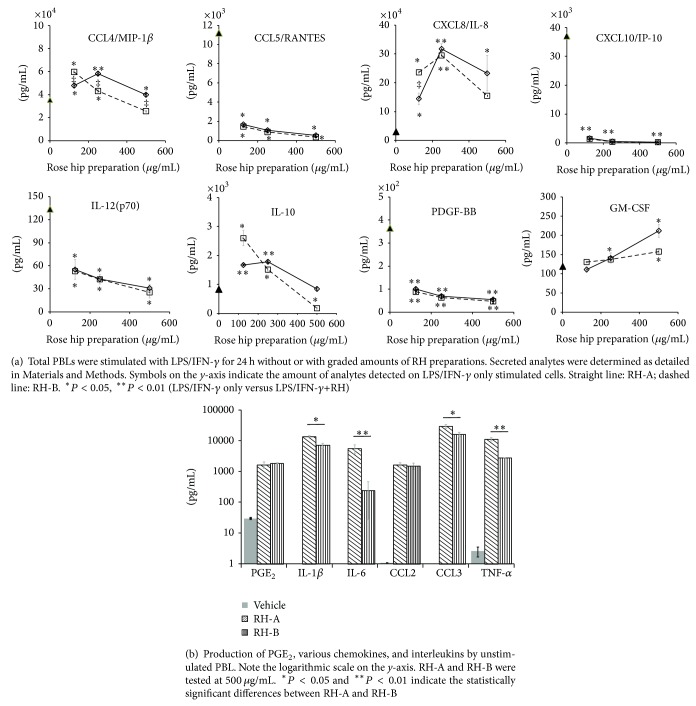
Effect of RH on production of PGE_2_ and cytokines by activated peripheral blood leukocytes.

We investigated the effect of rose hip preparations on cells of the peripheral blood, which is an obligatory passage for nutritional supplements to the target tissue (i.e., cartilage). LPS/IFN-*γ* treatment induced the secretion of large quantities of CCL2/MCP-1, CCL3/MIP-1*α*, CCL4/MIP-1*β*, CCL5/RANTES, CXCL10/IP-10, and CXCL8/IL-8 (Table 2 and Figure 1). Rose hip preparations significantly reduced the secretion of, for example, CCL5/RANTES, and CXCL10/IP-10. Conversely, CXCL8/IL-8 and CCL3/MIP-1*α* secretion were increased, while other chemokines (CCL11/eotaxin, CCL2/MCP-1, and CCL4/MIP-1*β*) were not markedly altered. Similarly the secretion of interleukins and cytokines was influenced by RH preparations: IL-6 and IL-12(p70) were less produced in the presence of increasing RHP amounts, while IL-10, IL-1*β*, and TNF-*α* were secreted at higher levels. The secretion of CCL5/RANTES, CXCL10/IP-10, and IL-12(p70) was influenced by the lowest tested concentration, whereas changes in others (e.g., CXCL8/IL-8, CCL3/MIP-1*α*, and IL-6) required high RHP concentrations. RH-B had more potent effects and thus a larger impact on inflammatory mediators than its RH-A counterpart (see Table 2). It should be mentioned that secretion of PGE_2_, CCL2/MCP-1, CCL4/MIP-1*β*, and cytokines (e.g., IL-1*β*, IL-6 and TNF-*α*) was stimulated by RH-A or RH-B even in the absence of LPS/IFN-*γ* (Figure 1). RH-A and RH-B did not impair cell viability (data not shown; see also [20]), since some of the cellular parameters were not altered by graded amount of the substances.

**Figure 2:**
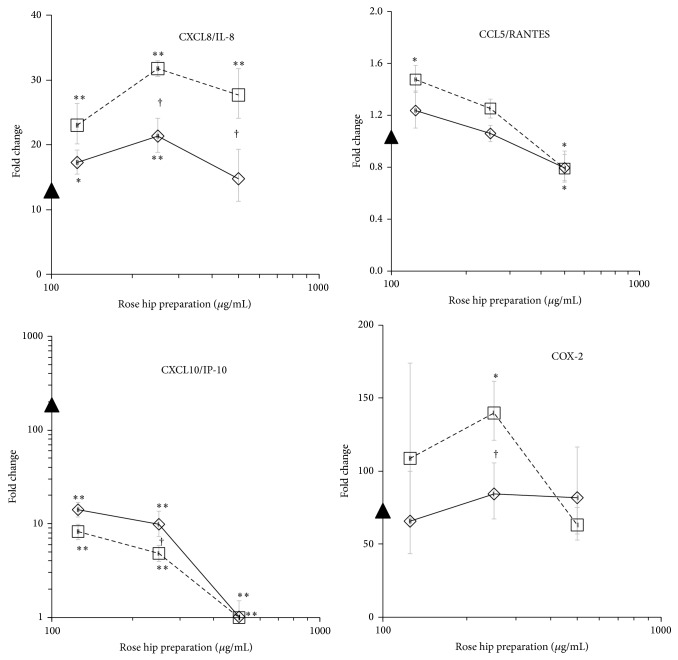
Effect of RH-A (straight line) and RH-B (dashed line) on gene expression in PBL. Cells were stimulated for 12 h with the indicated amount of substances and the gene expression was quantified by RT-PCR. Symbol on *y*-axis indicates the expression levels in LPS/IFN-*γ* only stimulated PBL.  ^*^ *P* < 0.05,  ^**^ *P* < 0.01 (LPS/IFN-*γ* only versus LPS/IFN-*γ*+RH). ^†^ *P* < 0.05 and ^‡^ *P* < 0.01 indicate the differences between RH-A and RH-B.

Next, we investigated whether RHP acted at the level of transcription or posttranscriptionally. Changes in gene expression levels showed a pattern which was similar to that of the respective proteins, as best illustrated for CXCL10/IP-10, IL-6, and CXCL8/IL-8 (Table 3 and Figure 2). In comparison to RH-A, the RH-B counterpart had a stronger impact on the expression of inflammatory genes. This suggests that RH-B and RH-A modulated the LPS/IFN-*γ*-induced changes in PBL at the level of transcription. One notable exception was CCL5/RANTES, which was released from activated PBL even though the respective gene activity was virtually unaltered. Also, IL-1*β* mRNA levels were decreased by RHP, whereas the cells discharged increased amounts of IL-1*β* (see Figure 1). RHP induced PGE_2_ in unstimulated PBL and increased it in LPS/IFN-*γ*-activated PBL (not shown; see also [20]). This might be explained by the presence of fatty acids that were converted into substrates for COX-1 in PBL.

### 3.3. Rose Hip Preparations Modulate Catabolic Gene Expression in Activated Normal Human Chondrocytes

**Figure 3:**
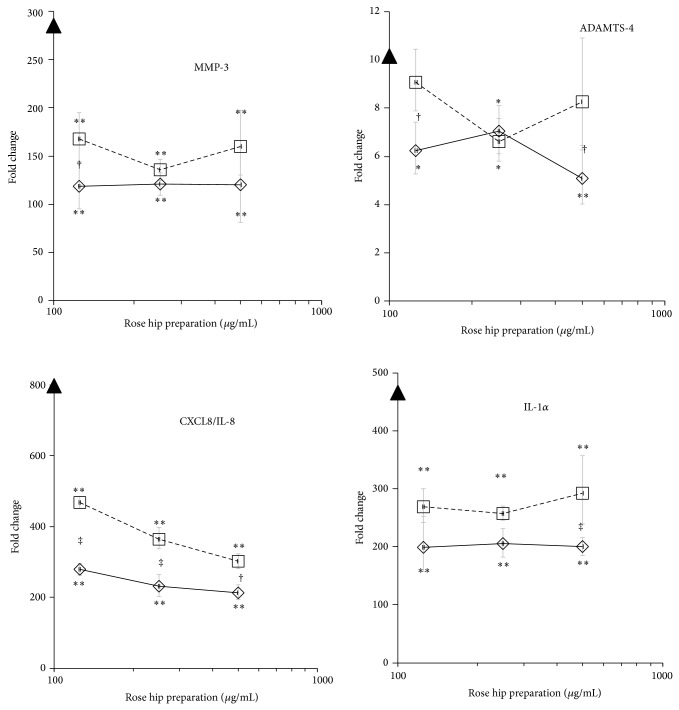
Effect of RH preparations on expression levels in human primary chondrocytes. NHAC-kn cells were stimulated with IL-1*β* for 4 h and the expression levels of indicated genes were quantified by RT-PCR. Triangles on* y*-axis indicate the expression levels in IL-1*β* only treated NHAC-kn. Straight line: RH-A; dashed line RH-B.  ^*^ *P* < 0.05,  ^**^ *P* < 0.01 (IL-*β* only versus IL-1*β* + RHP). ^†^ *P* < 0.05 and ^‡^ *P* < 0.01 indicate the differences between RH-A and RH-B.

Treatment of NHAC-kn with IL-*β* drastically increased expression levels of catabolic and inflammatory genes which reflect the induction of OA conditions in normal chondrocytes (Table 4 and Figure 3) [12]. Within 4 h of stimulation, IL-1*β* significantly upregulated catabolic genes (MMP-1, MMP-3, MMP-13, and ADAMTS-4), cytokine genes (IL-1*α*, IL-6, and TNF-*α*), and chemokines (CXCL8/IL-8, CCL5/RANTES, CXCL2/MIP-2, and CXCL20/MIP-3*α*). RH-A concentration-dependently reduced MMP-1 and MMP-3 expression by >50%, while MMP-13 expression was diminished by 90% at the highest dose of RH-A. ADAMTS-4 mRNA levels were also reduced by ~50%. Proinflammatory cytokine genes like IL-1*α* and IL-6 were significantly downregulated. With regard to chemokine genes (CXCL8/IL-8, CCL5/RANTES, CXCL2/MIP-2, and CXCL20/MIP-3*α*), RH-A mitigated their expression levels to a similar extent at 125–500 *μ*g/mL (up to 80% reduction). RH-B induced changes in gene expression that were comparable to those observed by RH-A, although there were quantitative differences in MMP-1, MMP-13, IL-1*α*, CXCL8/IL-8, and CCL-2/MIP-2: in general, RH-B exerted weaker effects than RH-A (see “*fold change*” in Table 4). This is in contrast to the observations made in PBL (Table 3). Notably, CCL5/RANTES gene expression was dramatically induced in IL-1*β* treated chondrocytes, but it was not upregulated in PBL. This observation points to cell- and tissue-specific modes of action of RHP and to distinct effects of RH-A and RH-B, respectively, in PBL and chondrocytes.

### 3.4. Comparison of Rose Hip Induced Changes of Variables in Different Cell Types

**Figure 4:**
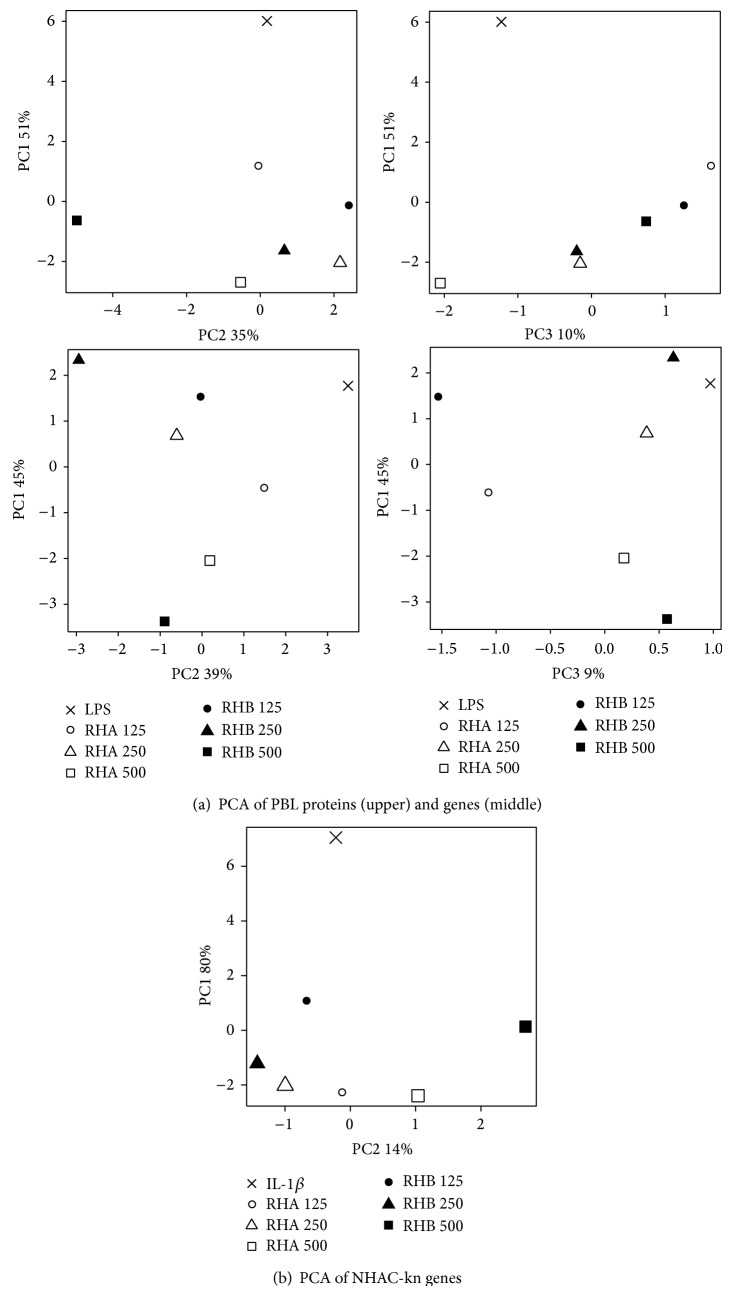
Principal component analysis showing the impact of different treatments and concentrations of RH-A and RH-B in peripheral blood leukocytes (upper panels) and normal human chondrocytes (bottom panel). PBLs were treated with LPS/IFN-*γ* alone or LPS/IFN-*γ* and RHP at the indicated concentrations (in *μ*g/mL). NHAC-kn were activated with IL-1*β* alone or IL-1*β* and RHP at the indicated concentrations.

In order to comprehensively assess the rose hip induced changes in OA biomarkers, we performed principal component analyses (PCA). PCA is a dimension reduction method that allows transforming a complex highly correlated dataset (e.g., all OA biomarkers) into a much smaller set of uncorrelated variables, that is, principal components (PCs). In our experimental data set, the PCs were taken as “meta-biomarker” and derived in decreasing order of importance, the first PC explaining most of the variation (and thus representing most of the information) contained in the original dataset. Plotting the OA biomarkers in PCA space allows to visualize and interpret these changes, which is very difficult in the high-dimensional setting of before. Three different PCAs were run, illustrating the global effects of RH-A and RH-B on PBLs at the protein and gene level and on NHAC-kn gene level. The PCA were based on the expression of 17 PBL proteins, 10 PBL genes, and 14 NHAC-kn genes, respectively. At the protein level, LPS/IFN-*γ*-activated PBL were clearly separated from the LPS/IFN-*γ*-activated/RHP-treated cells showing a treatment effect on PC1. Additionally a concentration-dependent effect for RH-A could be observed for PC1 as well. Concentration-dependent effects for RH-B were mainly visualized by principal component 2 (Figure 4). At the gene level, LPS/IFN-*γ*-activated PBL were separated from higher dose LPS/IFN-*γ*-activated/RHP-treated cells within the first principal component. A similar clustering of treatments indicating a treatment effect was also observed within PC2. Additionally, RH-B exerted a more discriminating effect than RH-A within PC2. The cumulative explained variability (CEV) (explained variation of PC1 + PC2 + PC3) for PBL at the protein and gene level was 96% and 94%, respectively, with PC1 explaining 51% and 45% of the variability. For NHAC-kn, CEV was 94% for variations observed at the gene level (PC1 + PC2, PC1 by itself explaining 80%). An effect of treatment was observable and mostly reflected by PC1, whereas the effect of substance concentrations (especially contrasting the high dose from the other doses) was mainly distinguished by principal component 2. A stronger effect of RH-B as compared to RH-A could also be observed in PC1. Taken together, the data indicate a distinguishable and beneficial effect of RH on OA biomarkers with RH-B being a stronger discriminator than RH-A.

## 4. Discussion

In this study, a panel of biological activities of rose hip powder prepared from the whole fruit or from shells has been compared. It provides evidence that cellular features related to inflammatory responses and cartilage destruction were modulated by the complex mixture of substances contained in RHP (see Table 1). These have been measured in murine and human cellular models and are fully consistent with those previously reported [20]. In LPS-activated murine macrophages RHP exhibited mainly anti-inflammatory properties including reduced production of nitric oxide, COX-2 dependent PGE_2_, various chemokines, and proinflammatory cytokines [20]. In PBL, the expression of interleukins and cytokines were mostly mitigated by RHP, while growth factor production like GM-CSF and G-CSF were increased. We observed a dichotomy with regard to chemokine expression: CCL5/RANTES, CCL11/eotaxin, and CXCL10/IP-10 drastically decreased, whereas others augmented in the presence of RHP (e.g., CXL8/IL-8, CCL3/MIP-1*α*, and CCL4/MIP-1*β*). With regard to CCL5/RANTES and CXCL8/IL-8, their cellular release was modulated by RHP, while gene expression was not altered. Therefore, RHP could contribute to improve the amplitude and length of the acute inflammatory response through a more vigorous mobilization of lymphocytes and macrophages, while it dampens an excessive cell mobilization in chronic inflammation processes like in OA. This needs to be investigated in further experiments, where RHP should be tested on distinct subpopulations of PBL such as neutrophils, monocytes, and lymphocytes.

The pattern of observed* in vitro* activities was more definite in the target tissue cell equivalent, that is, chondrocytes, where gene expression of CCL5/RANTES, CXCL10/IP-10, CXCL2/MIP-2, and CCL20/MIP-3*α* was reduced by RHP. Concomitantly, the expression of genes that degrade ECM was diminished and thus reflected a chondroprotective effect of RHP on cartilage tissue. Previous studies highlighted the importance of chemokines in OA [27], with CCL5/RANTES and CXCL8/IL-8 being activated in chondrocytes [9–11, 28]. Likewise, IL-1*β* activation robustly modified chemokine and chemokine receptor expression in chondrocytes [12]. The results of the present study corroborate previous data obtained with another source of RHP [20].

It is premature to unambiguously define the bioactive constituents of RH-A and RH-B, whose composition considerably differed (Table 1). Removing seeds from rose hip preparations resulted in higher contents of anti-inflammatory substances like betulinic acid, ursolic acid, galactolipids, EPA, and DHA. On the other side, seeds contained the bulk of fatty acids which could serve as substrate for inflammatory mediators such as PGE_2_. The multiple measured effects of RHP in different cells have been compared by PCA. These analyses demonstrated that RH-A and RH-B robustly modified a pattern or cluster of biological responses, as anticipated for beneficial effects of nutritional supplements (reviewed by, e.g., [29]). More importantly, two of the three PCAs performed (at the level of PBL and NHAC-kn genes) showed that RH-B exhibited an overall stronger effect on OA biomarkers than RH-A. Given the demonstrated health benefit of rose hip in clinical studies (for review see, e.g., [17]), we hypothesize that in clinical studies RH-B is more efficacious or can be used in lower doses than RH-A in the treatment of OA conditions. Indeed, a recently completed randomized active-controlled trial provided evidence that, taken at half the original dose, the novel enhanced rosehip powder (RH-B) had similar effects in patients with symptomatic OA as the original rosehip product (RH-A) [30].

## 5. Conclusions

A new form of rosehip powder has enhanced* in vitro* anti-inflammatory and chondroprotective properties in human peripheral blood leukocytes and primary chondrocytes.

## Figures and Tables

**Table 1:** Constituents of RH-A and RH-B.

Constituent [mg/kg]	RH-A (with seeds)	RH-B (without seeds)
GLGPG^1^	114	210
MGDG^2^	71	120
DGDG^3^	1220	1900
Betulinic acid^4^	662	1078
Betulinic, oleanolic, and ursolic acid	1060	1711
Vitamin C	3146	3182
Vitamin E	129	212
Total carotenoids	190	250
*β*-Carotene	36	36
Lycopene	89	137
Total fatty acid	32810	7360
Linoleic acid	16390	2230
EPA	<LOD^5^	80
DHA	<LOD	60

^1^Galacto-lipid (2S)-1, 2-di-O-[(9Z, 12Z, 15Z)-octadeca-9, 12, 15-trienoyl]-3-O-*β*-D-galactopyranosyl glycerol.

^2^Mono-galactosyl diglyceride.

^3^Di-galactosyl diglyceride.

^4^Betulinic acid, oleanolic acid, and ursolic acid were determined according to a validated in-house method; vitamin C and vitamin E were analyzed according to the official methods EN14130 and EN12822, respectively; linoleic acid, EPA, and DHA were measured according to the official method of ISO 12966-2.

^5^Limits of detection.

**Table 2:** Secretion of proteins by activated PBL. LPS/IFN-*γ*-stimulated cells were cultured with 125–500 *μ*g/mL RHP for 24 h and proteins were quantified by multiparametric analysis. Only values for (LPS/IFN-*γ*-stimulated) cells and (500 *μ*g/mL RHP + LPS/IFN-*γ*-stimulated) cells are given. Differences between RH-A and RH-B are given in the right-side column.

Protein	LPS/IFN-*γ*	LPS/IFN-*γ* + RH-A (500 *μ*g/mL)		LPS/IFN-*γ* + RH-B (500 *μ*g/mL)		RH-A versus RH-B
	pg/mL ± stdev	pg/mL ± stdev	*P* value^a^	pg/mL ± stdev	*P* value	
CCL11/Eotaxin	194 ± 7	150 ± 16	*0.069 *	141 ± 15	*0.045 *	NS^b^
CCL2/MCP-1	1136 ± 126	1513 ± 145	*0.109 *	837 ± 106	*0.123 *	0.042
CCL3/MIP-1*α*	4028 ± 513	13050 ± 919	*0.007 *	5455 ± 495	*0.105 *	0.010
CCL4/MIP-1*β*	35500 ± 1697	93875 ± 1167	*0.095 *	25800 ± 7354	*0.211 *	0.009
CXCL8/IL-8	30600 ± 3253	233250 ± 61165	*0.042 *	155125 ± 74635	*0.142 *	NS
CCL5/RANTES	11188 ± 1962	545 ± 91	*0.017 *	353 ± 54	*0.016 *	NS
CXCL10/IP-10	37075 ± 11208	266 ± 27	*0.043 *	228 ± 35	*0.043 *	NS

IL-1*β*	7165 ± 1520	37800 ± 1980	*0.003 *	18550 ± 1697	*0.019 *	0.021
IL-6	59975 ± 10006	47000 ± 6293	*0.261 *	6525 ± 92	*0.017 *	0.003
IL-12 (p70)	134 ± 17	31 ± 5	*0.015 *	25 ± 7	*0.015 *	NS
IL-10	778 ± 84	845 ± 73	*0.487 *	179 ± 16	*0.010 *	0.010
TNF-*α*	10515 ± 474	27325 ± 3712	*0.024 *	6745 ± 983	*0.039 *	0.044
GM-CSF	113 ± 10	212 ± 17	*0.018 *	158 ± 8	*0.040 *	NS
G-CSF	831 ± 165	14075 ± 2510	*0.018 *	1272 ± 16	*0.064 *	0.005

^a^ *P* value: significance value between (LPS/IFN-*γ*) and (LPS/IFN-*γ* + substance) treatment.

^b^NS: not significant.

**Table 3:** Effects of RHP on gene expression in stimulated PBL (cultured for 12 h). LPS/IFN-*γ*-stimulated cells were cultured with 125–500 *μ*g/mL RHP for 12 h and gene expression was quantified by RT-PCR. Fold changes were calculated as indicated in Materials and Methods. Values for (LPS/IFN-*γ*-stimulated) cells and (500 *μ*g/mL RHP + LPS/IFN-*γ*-stimulated) cells are given.

Gene	LPS/IFN-*γ*	LPS/IFN-*γ* + RH-A (500 *μ*g/mL)		LPS/IFN-*γ* + RH-B (500 *μ*g/mL)		RH-A versus RH-B
	Fold change	Fold change	*P* value^a^	Fold change	*P* value	
COX-2	83	107	0.061	81	0.739	NS^b^
TNF-*α*	83	55	0.006	42	0.001	NS
IL-1*α*	3513	1347	<0.001	859	<0.001	NS
IL-1*β*	1460	538	<0.001	410	<0.001	NS
IL-6	15928	9261	0.058	3656	<0.001	0.04

CCL5/RANTES	1	0.8	0.070	0.8	0.054	NS
CXCL8/IL-8	13	15	0.418	28	<0.001	0.03
CXCL10/IP-10	187	1	<0.001	<LOD^c^	<0.001	Not applicable
CXCL2/MIP-2	12	22	0.012	25	0.002	NS
CCL20/MIP-3*α*	3634	5454	0.014	6738	0.014	NS

^a^ *P* value: significance value between (LPS/IFN-*γ*) and (LPS/IFN-*γ* + substance) treatment.

^b^NS: not significant.

^c^Below level of detection.

**Table 4:** Effects of RHP on catabolic gene expression in human primary chondrocytes. IL-1*β*-stimulated cells were cultured with 125–500 *μ*g/mL RHP for 4 h and gene expression was quantified by RT-PCR. Fold changes were calculated as indicated in Materials and Methods. Values for (IL-1*β* stimulated) cells and (500 *μ*g/mL RHP + IL-1*β*) stimulated cells are given.

Gene	IL-1*β*	IL-*β* + RH-A (500 *μ*g/mL)		IL-1*β* + RH-B (500 *μ*g/mL)		*P* value RH-A versus RH-B
	Fold change	Fold change	*P* value^a^	Fold change	*P* value	
MMP-1	28	16	0.001	26	0.620	0.032
MMP-3	286	120	0.001	160	0.003	NS^b^
MMP-13	1.7	0.2	<0.001	0.5	<0.001	0.040
ADAMTS-4	10	5	0.006	8	0.295	NS
CXCL2/MIP-2	467	122	<0.001	180	<0.001	0.091
CXCL20/MIP-3*α*	642	253	0.001	331	0.010	NS
CCL5/RANTES	176	127	0.008	151	0.241	NS
CXCL8/IL-8	797	214	<0.001	301	<0.001	0.087
IL-1*α*	466	200	0.001	292	0.032	NS
IL-6	1454	897	0.022	746	0.006	NS
TNF-*α*	1120	1081	0.727	1704	0.005	NS

^a^ *P* values: significance value between (IL-1*β*) and (IL-1*β* + substance) treatment.

^b^Not significant.

## Abbreviations

IL: Interleukin
LPS: Lipopolysaccharide
NO: Nitric oxide
MMP: Matrix metalloproteinase
OA: Osteoarthritis
PBL: Peripheral blood leukocytes
PGE_2_: Prostaglandin E_2_
RH: Rose hip
RHP: Rose hip powder.
